# A network pharmacology approach to explore active compounds and pharmacological mechanisms of a patented Chinese herbal medicine in the treatment of endometriosis

**DOI:** 10.1371/journal.pone.0263614

**Published:** 2022-02-07

**Authors:** Yan Wu, Yuhang Zhu, Ningning Xie, Hui Wang, Fangfang Wang, Jue Zhou, Fan Qu

**Affiliations:** 1 Women’s Hospital, School of Medicine, Zhejiang University, Hangzhou, Zhejiang, China; 2 Rehabilitation & Sports Medicine Research Institute of Zhejiang Province, Zhejiang Provincial People’s Hospital, People’s Hospital of Hangzhou Medical College, Hangzhou, Zhejiang, China; 3 College of Food Science and Biotechnology, Zhejiang Gongshang University, Hangzhou, Zhejiang, China; Alagappa University, INDIA

## Abstract

**Objective:**

Endometriosis is a common benign disease in women of reproductive age. Qu’s formula (QUF) is a patented Chinese herbal medicine for treating endometriosis that has been proven to be effective in treating and preventing the recurrence of endometriosis. This study is aimed to discover its molecular mechanism and to explore the potential drug targets.

**Methods:**

A QUF target and endometriosis-related gene set was identified by the Traditional Chinese Medicine Systems Pharmacology (TCMSP) and Bioinformatics Analysis Tool for Molecular Mechanism of Traditional Chinese Medicine (BATMAN-TCM) databases and five disease-gene databases. Gene ontology (GO) and Kyoto Encyclopedia of Genes and Genomes (KEGG) enrichment analyses were performed, and a protein–protein interaction (PPI) network was established to discover the potential mechanism. MalaCards was searched for targets and signaling pathways related to endometriosis, and the search results were also used to identify the key factors in QUF. Molecular docking was performed to visualize the interactions between the effective molecules and proteins encoded by critical genes. Cell experiments and molecular dynamics (MD) simulations were used to further validate the therapeutic effects of the active compounds in QUF on endometriosis.

**Results:**

A compound-target network with 117 nodes (94 genes and 23 active compounds) and 224 edges was generated. The results of GO and KEGG analyses indicated that QUF could act by regulating the immune response, apoptosis and proliferation, oxidative stress, and angiogenesis. *VEGFA*, *CXCL8*, *CCL2*, *IL1B* and *PTGS2* were selected for molecular docking analysis from two critical subnetworks with high correlation scores in MalaCards, and the active compounds of QUF had binding potential and high affinity for them. The mRNA expression levels of *CCL2*, *IL1B* and *PTGS2* significantly decreased after treatment with quercetin. MD simulations showed that the combinations of quercetin and these proteins were relatively stable.

**Conclusion:**

The network pharmacological strategy integrates molecular docking to unravel the molecular mechanism by which QUF protects against endometriosis. Our findings not only confirm the clinical effectiveness of QUF but also provide a foundation for further experimental study.

## Introduction

Endometriosis is a common gynecological disease characterized by the presence of endometrial-like tissue outside the uterus [1]. This estrogen-dependent, benign, inflammatory disease can cause dysmenorrhea, dyspareunia, chronic pain, and infertility [2]. It is estimated to affect 6%–10% of women of reproductive age [3]. Current interventions for endometriosis consist of surgical removal of lesions and hormonal medication, often with limited efficacy and unacceptable side effects [4,5]. Moreover, approximately 50% of women with endometriosis have recurrent symptoms over 5 years [6].

Traditional Chinese medicine (TCM) has been widely used to treat gynecological diseases for centuries. Guizhi Fuling Wan (GZFLW) is a classical prescription for endometriosis treatment in TCM [7]. A recent network pharmacology analysis indicated that GZFLW can treat endometriosis through multiple mechanisms [8]. Animal experiments have shown that GZFLW can attenuate endometriosis in rats by immunological regulation and induction of apoptosis [9,10].

Qu’s formula (QUF) is a patented Chinese herbal medicine for treating endometriosis; it consists of four herbs: Common Lophatherum (Danzhuye), Radix Paeoniae Rubra (Chishao), Curcumae Rhizoma (Ezhu), and Radix Bupleuri (Chaihu) (Chinese National Invention Patent CN:201410824487:A). A prospective, multicenter and controlled trial suggested that QUF could prevent the recurrence of endometriosis and decrease the serum levels of cancer antigen 125 (CA-125) and interleukin 18 (IL-18) [11]. Moreover, no side effects were reported during the research and the follow-up period [11]. However, its mechanism of action is unclear due to the complex composition.

With the development of network pharmacology, our understanding of the multiple mechanisms of action of drugs has greatly increased [12]. The establishment of a TCM network pharmacology approach helps us to discover TCM from a systems perspective and at the molecular level. It could change the research paradigm from "one target, one drug" to a new "network target, multicomponents" mode [13].

**Fig 1:**
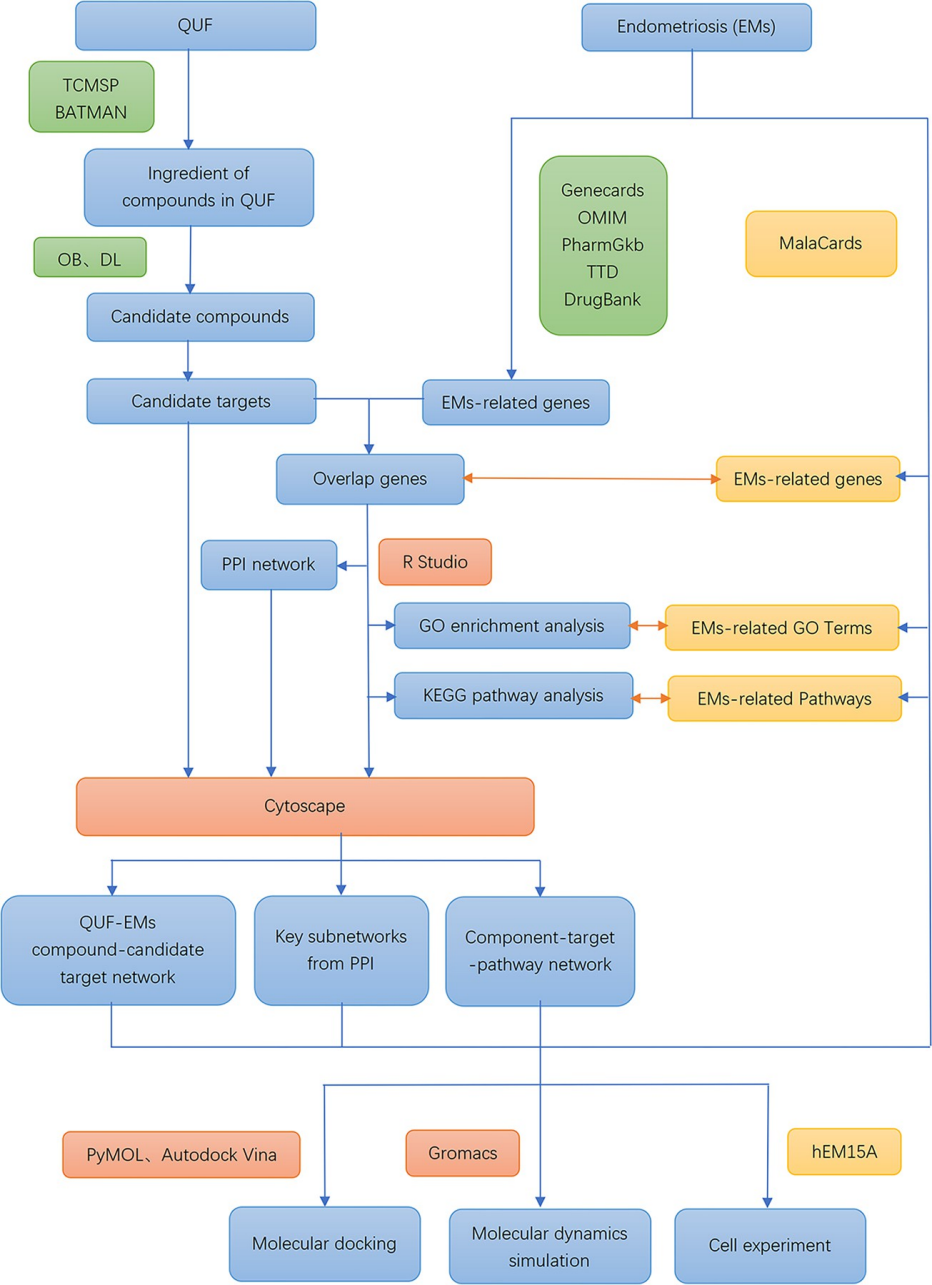
Workflow for Qu’s formula (QUF) against endometriosis. Abbreviations: QUF: Qu’s formula; TCMSP: Traditional Chinese Medicine Systems Pharmacology; BATMAN: Bioinformatics Analysis Tool for Molecular Mechanism; OB: Oral Bioavailability; DL: Drug-Like; EMs: Endometriosis; PPI: Protein–Protein Interaction.

In the present study, we used a network pharmacology approach to explore the potential mechanism of action for QUF in treating endometriosis. We first obtained the QUF targets and the endometriosis-related gene set from the databases. Then, we performed a network analysis of the overlapping genes. We also searched the human disease database MalaCards for targets and signaling pathways related to endometriosis. In addition, we performed docking studies to predict the interactions that allow the main compounds in QUF to bind to the key targets. Finally, cell experiments and molecular dynamics (MD) simulations were conducted to validate the interaction between the active compounds in QUF and key target proteins in endometriosis. The workflow of the network pharmacology approach is shown in Fig 1.

## Materials and methods

### Identification of active compounds and related targets in QUF

Traditional Chinese Medicine Systems Pharmacology (TCMSP, https://tcmspw.com/tcmsp.php) is a Chinese medicine pharmacology database, that contains information about the herbs used in TCM. It provides information on the absorption, distribution, metabolism and excretion (ADME) characteristics of the individual compounds, their targets, related diseases, and pathways. It was first obtain the main compounds of QUF. The Bioinformatics Analysis Tool for Molecular Mechanism of Traditional Chinese Medicine (BATMAN-TCM, http://bionet.ncpsb.org/batman-tcm/index.php), another bioinformatics analysis tool for identifying the main components of TCM, was used as a supplementary database when an herb could not be found in TCMSP (e.g. Common Lophatherum).

We searched for the herbs in QUF (Radix Paeoniae Rubra, Curcumae Rhizoma, and Radix Bupleuri) in TCMSP, and filtered active compounds by the criteria of the oral bioavailability (OB) ≥ 30% and a drug-like (DL) index ≥ 0.18. If the herb compounds were obtained by BATMAN-TCM (Common Lophatherum), their structure was first downloaded from PubChem (https://pubchem.ncbi.nlm.nih.gov) and then imported into SwissADME (http://www.swissadme.ch) to obtain the pharmacokinetics and druglikeness parameters. As an active compound, gastrointestinal (GI) absorption should be “High” and the number of “YES” in the druglikeness field should be greater than or equal to two.

For each active compound, we searched for related target genes in TCMSP or BATMAN-TCM. The target gene set of QUF was acquired after gene symbol annotation with the help of UniProt (https://www.uniprot.org).

### Prediction of target genes associated with endometriosis

We searched five databases, including the Genecards database (https://www.genecards.org/), OMIM database (https://omim.org/), PharmGkb database (https://www.pharmgkb.org/), TTD database (http://db.idrblab.net/ttd/), and DrugBank database (https://www.drugbank.ca/), with the key word “Endometriosis” to obtain endometriosis-related genes. We established an endometriosis-related gene set by combining the search results.

### Establishment of a QUF-endometriosis gene network

The drug target and disease-related gene set was obtained by intersecting the QUF target gene set with the endometriosis-related gene set. The compound-target visual interaction network was established by Cytoscape version 3.8.2 based on the overlapping genes. In such a network, a node can represent a compound or a gene/protein, and an “edge” is an association between the nodes. Hubs with high centrality are considered key hubs in the network.

### Biological function analysis

Gene Ontology (GO) and Kyoto Encyclopedia of Genes and Genomes (KEGG) pathway enrichment analyses were used to reveal the underlying biological processes (BP), cellular components (CC), molecular functions (MF) and key signaling pathways. We set the filter as the adjusted P value <0.05 and q-value < 0.05 and performed the enrichment analysis with R software version 4.0.3 (Bioconductor, clusterProfiler) [14].

### Protein–protein interaction (PPI) network and critical subnetwork

The overlapping target proteins of endometriosis and QUF were used to construct a protein–protein interaction (PPI) network with multiple protein patterns on the Search Tool for the Retrieval of Interacting Genes/Proteins (STRING) platform (https://string-db.org). We set the parameter as moderate confidence (0.400) and downloaded the “string_interactions.tsv” file.

Then we imported the downloaded file into Cytoscape to investigate the critical subnetwork. In detail, we calculated the primary score of betweenness, closeness, degree, eigenvector, LAC, and network scores by the CytoNCA plugin and filtered genes according to the criteria that each score was higher than the median value. The filtered genes were used to construct a primary subnetwork. Then, the filter process was conducted again to acquire the final critical subnetwork. The CytoHubba plugin in Cytoscape was another method we used to analyze the top 18 genes in the PPI network and to construct the critical subnetwork.

### MalaCards search

MalaCards is an integrated database of human diseases and their annotation. It can analyze disease-related gene sets in GeneDecks to generate the related pathways, phenotypes, compounds and GO terms and sort them by composite correlation scores [15]. MalaCards was searched with “endometriosis” as the keyword and then genes, signaling pathways, and other pieces of information related to endometriosis were shown.

### Construction of a component-target-pathway network

The genes highly related to endometriosis in MalaCards were selected to build the component-target-pathway network. The integrated network was constructed using Cytoscape 3.8.2 to identify the relationships between protein targets and each compound and the involved pathways.

### Molecular docking technology

Genes from two critical subnetworks with high composite correlation scores in MalaCards were selected for molecular docking analysis. The 3-dimensional (3D) structure of the receptor proteins encoded by the selected genes (PDB ID: 3QTK, 5WDZ, 3IFD, 5I1B and 5F1A) was downloaded from the RCSB PDB database (https://www.rcsb.org). Additionally, the 2D structure for the molecule ligands was downloaded from the PubChem database (https://pubchem.ncbi.nlm.nih.gov). ChemBio 3D software was used to create 3D chemical structures and minimize their energy. PyMOL 2.4.1 software was used to perform the ligand and water removal of the receptor protein and AutoDock software was used to carry out hydrogenation and the charge calculation of proteins. Parameters of the receptor protein docking site were set to include the active pocket sites, where small molecule ligands bind. Finally, AutoDock Vina was used to dock the receptor protein with the small molecule ligands of the active compounds of QUF. The docking score was used to evaluate the protein–ligand binding potential. The results with a value ≤ -5 were selected and considered to have moderate binding potential and a tight connection. In addition, the mode of action of active compounds with the target proteins was analyzed using PyMOL software.

### Cell culture and treatment

The endometriosis immortalized eutopic endometrial stromal cell line (hEM15A) was purchased from the Chinese Centre for Type Cultures Collections (Wuhan, China). These cells were cultured in DMEM-H/F12 medium (Biological Industries, Israel) containing 10% FBS (Gibco, USA) and 100 U/mL penicillin/streptomycin (Biological Industries, Israel) at 37°C in a humidified atmosphere with 5% CO2.

The cells were seeded into 6-well plates at a density of 1 × 10^5^ cells per well. When hEM15A cells grew to 70% confluence, they were treated with 10 μM quercetin (Sigma, USA) or an equal volume of dimethyl sulfoxide (DMSO) (Solarbio, China) for 24 h.

### Reverse transcription and real-time quantitative polymerase chain reaction (rt-qPCR)

Total RNA was extracted using TRIzol reagent (Invitrogen, USA) and then reverse transcribed into cDNA with the PrimeScript™ RT reagent Kit with gDNA Eraser (Takara, Japan). Rt–qPCR was performed with an Applied Biosystems® ViiA™ 7 Real-Time PCR System (Life Technologies, USA) using a TB Green® Premix Ex Taq™ kit (Takara, Japan). The amplification included 40 PCR cycles of 95°C for 30 s, 95°C for 5 s and 60°C for 30 s. The specific primers for PCR amplification were synthesized by Generay (Shanghai, China) and the primer sequences are listed in Table 1. The relative mRNA expression was calculated by the 2−ΔΔCT method and compared to an internal control of beta-actin (β-actin).

**Table 1:** Primer sequences.

Gene	Forward primer	Reverse primer
***beta-actin***	CGACAGGATGCAGAAGGAG	ACATCTGCTGGAAGGTGGA
***VEGFA***	AGGGCAGAATCATCACGAAGT	AGGGTCTCGATTGGATGGCA
***CXCL8***	ACTGAGAGTGATTGAGAGTGGAC	AACCCTCTGCACCCAGTTTTC
***CCL2***	CAGCCAGATGCAATCAATGCC	TGGAATCCTGAACCCACTTCT
***IL1B***	TTCGACACATGGGATAACGAGG	TTTTTGCTGTGAGTCCCGGAG
***PTGS2***	ATGCTGACTATGGCTACAAAAGC	TCGGGCAATCATCAGGCAC

### Molecular dynamics (MD) simulation

The molecular dynamics simulation study was performed using Gromacs 2019.06 software to evaluate the stability and interaction of target proteins and the active compounds [16]. The Amber 99SB-ILDN force field was used to process the proteins and ligands, and the ligand topology was generated by ACPYPE Server (https://www.bio2byte.be/acpype/). A dodecahedron box was chosen, and a distance of 1.0 nm was set between the box edges and protein complex. The TIP3P water model was used to solve each system followed by neutralization with the appropriate amount of Na^+^ and Cl^−^. After the system energy was minimized, the system was controlled by NVT and NPT at a temperature of 300 K and a pressure of 101.325 kPa. After energy minimization and system equilibration, an MD simulation was executed without any restraint for 100 ns. Root mean square deviation (RMSD), root mean square fluctuation (RMSF), radius of gyration (Rg), and hydrogen bonds were used to evaluate the interaction between ligand and receptor in dynamics analysis.

## Results

### Screening of active compounds and potential targets

After screening the key ingredients of QUF in TCMSP, we obtained 549 main compounds, including 81 compounds in Curcumae Rhizoma, 119 compounds in Radix Paeoniae Rubra, and 349 compounds in Radix Bupleuri. Among them, 49 compounds passed the filters of OB ≥ 30% and DL ≥ 0.18, and a total of 530 targets were identified for these compounds. For Common Lophatherum, only four compounds (friedelin, cymarin, arundoin, cylindrin) were found in BATMAN-TCM. However, the GI absorption of these four compounds in SwissADME was “Low”. The pharmacokinetic properties of the active compounds and the corresponding number of targets are shown in S1 Table. Among them, quercetin was associated with 78 targets related to endometriosis and could be considered the most important compound in QUF.

**Fig 2:**
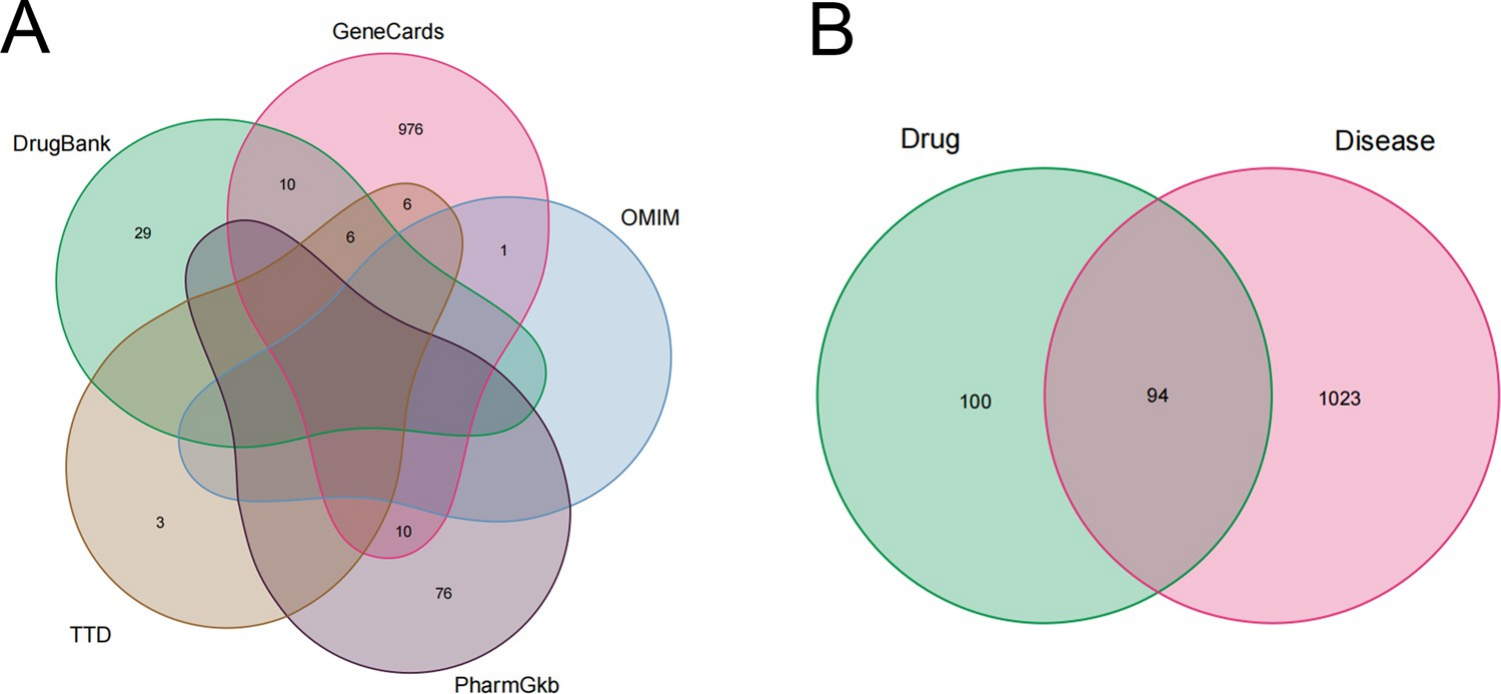
Identification of the drug-target interactions. (A) Identification of endometriosis-related genes by combining all the results from 5 databases. (B) Identification of the drug-target disease-related genes by taking an intersection of drug target genes and endometriosis-related genes.

In addition, we obtained 1009, 1, 86, 15 and 45 endometriosis-related genes from the Genecards, OMIM, PharmGkb, TTD, and DrugBank databases, respectively. After removing duplicates and combining the search results, a total of 1117 endometriosis-related genes were included in the acquired gene set (Fig 2A). In addition, by intersecting the compound-target genes and disease-related genes, we finally obtained the QUF target and endometriosis-related gene set (Fig 2B).

### Compound-target network

**Fig 3:**
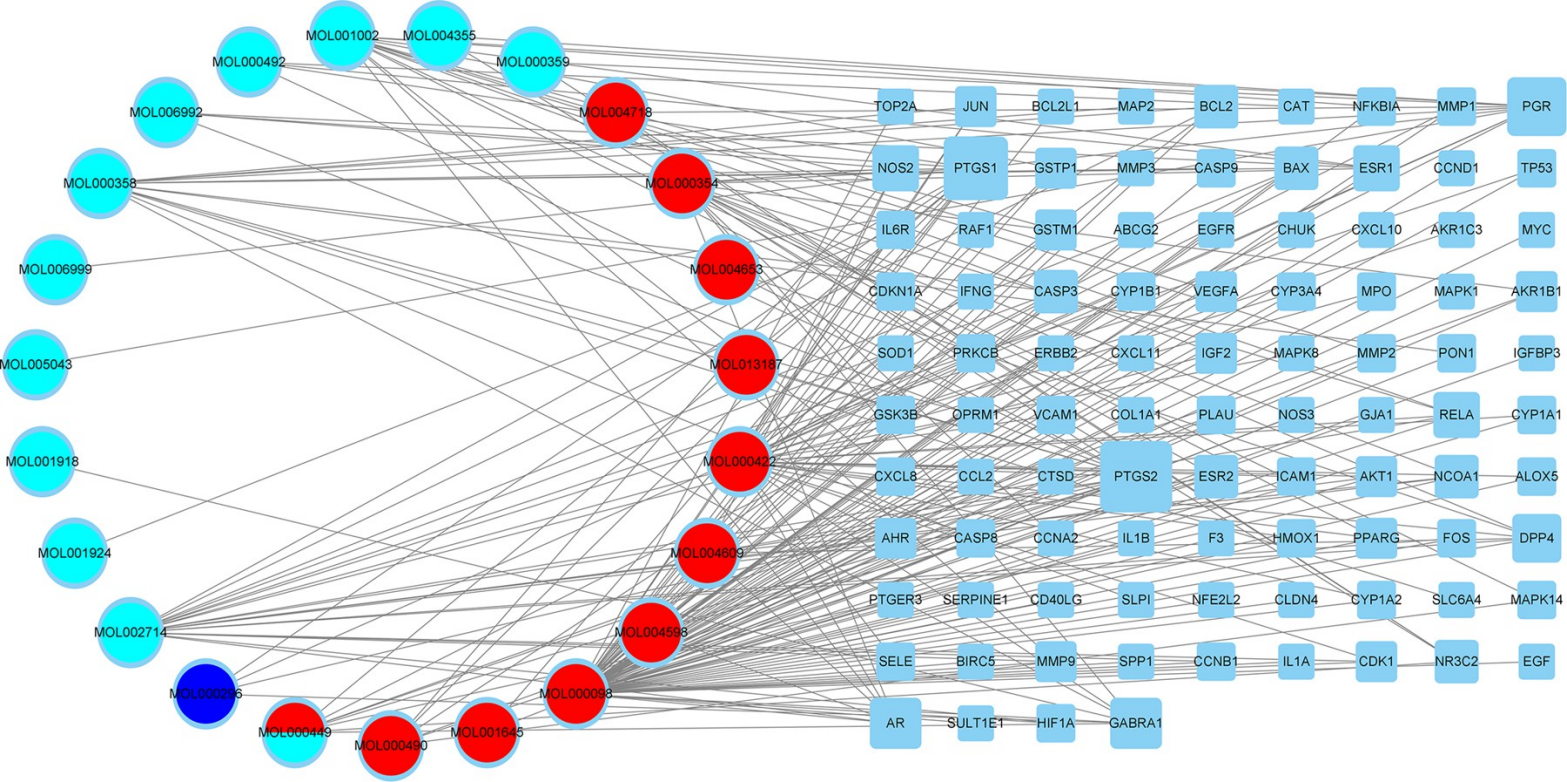
The drug-target interaction pharmacology network. Circles represent the small molecule active compounds in QUF (red: Radix Bupleuri; blue: Curcumae Rhizoma; aqua: Radix Paeoniae Rubra). Rounded rectangles represent endometriosis-related target genes, and edges represent the interaction between small molecule compounds and target genes.

After discovering the compound-target disease-related genes, we used Cytoscape 3.8.2 to visualize a compound-target interaction network with 117 nodes and 224 edges (Fig 3). Generally, one gene can be targeted by multiple active compounds, and one compound can target multiple genes. Among the 94 genes identified, the PTGS2 gene is targeted the most by the QUF ingredients.

### GO and KEGG enrichment analysis

**Fig 4:**
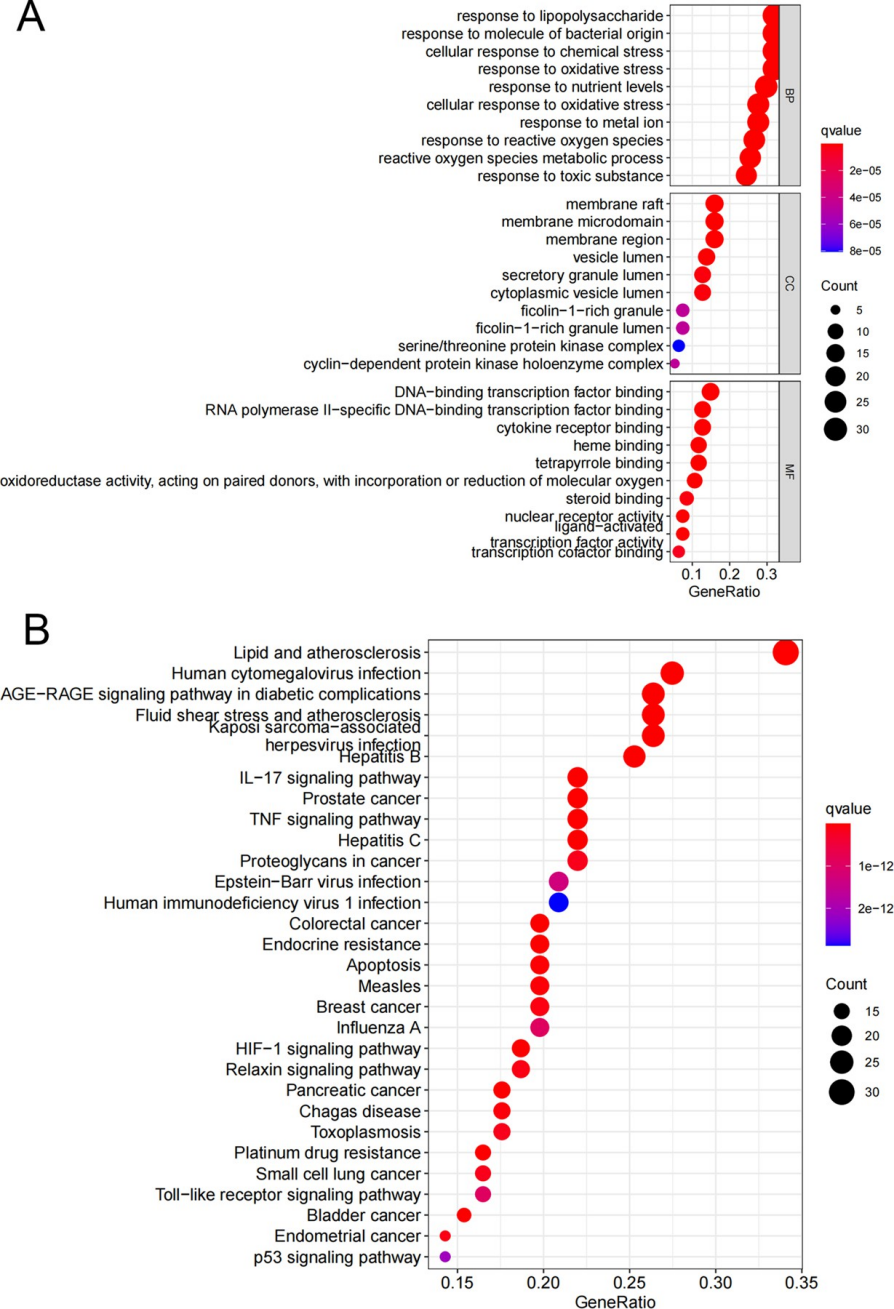
GO and KEGG enrichment analysis. (A) GO enrichment analysis of the target genes. (B) KEGG enrichment analysis of the target genes. The gene ratio refers to the ratio of enriched genes to all target genes, and counts refer to the number of enriched genes.

In the GO enrichment analysis, we obtained 2123 significantly enriched GO terms. The top 10 terms are shown in Fig 4A. The top five BP terms were response to lipopolysaccharide, response to molecule of bacterial origin, cellular response to chemical stress, response to reactive oxygen species, and response to oxidative stress. The top five CC terms were membrane raft, membrane microdomain, membrane region, vesicle lumen, and secretory granule lumen. The top five MF terms were heme binding, tetrapyrrole binding, DNA-binding transcription factor binding, nuclear receptor activity, and ligand-activated transcription factor activity. A total of 166 KEGG pathways were significantly enriched, and the bubble plot of the 30 most significant KEGG pathways is shown in Fig 4B.

### PPI network and core subnetwork

**Fig 5:**
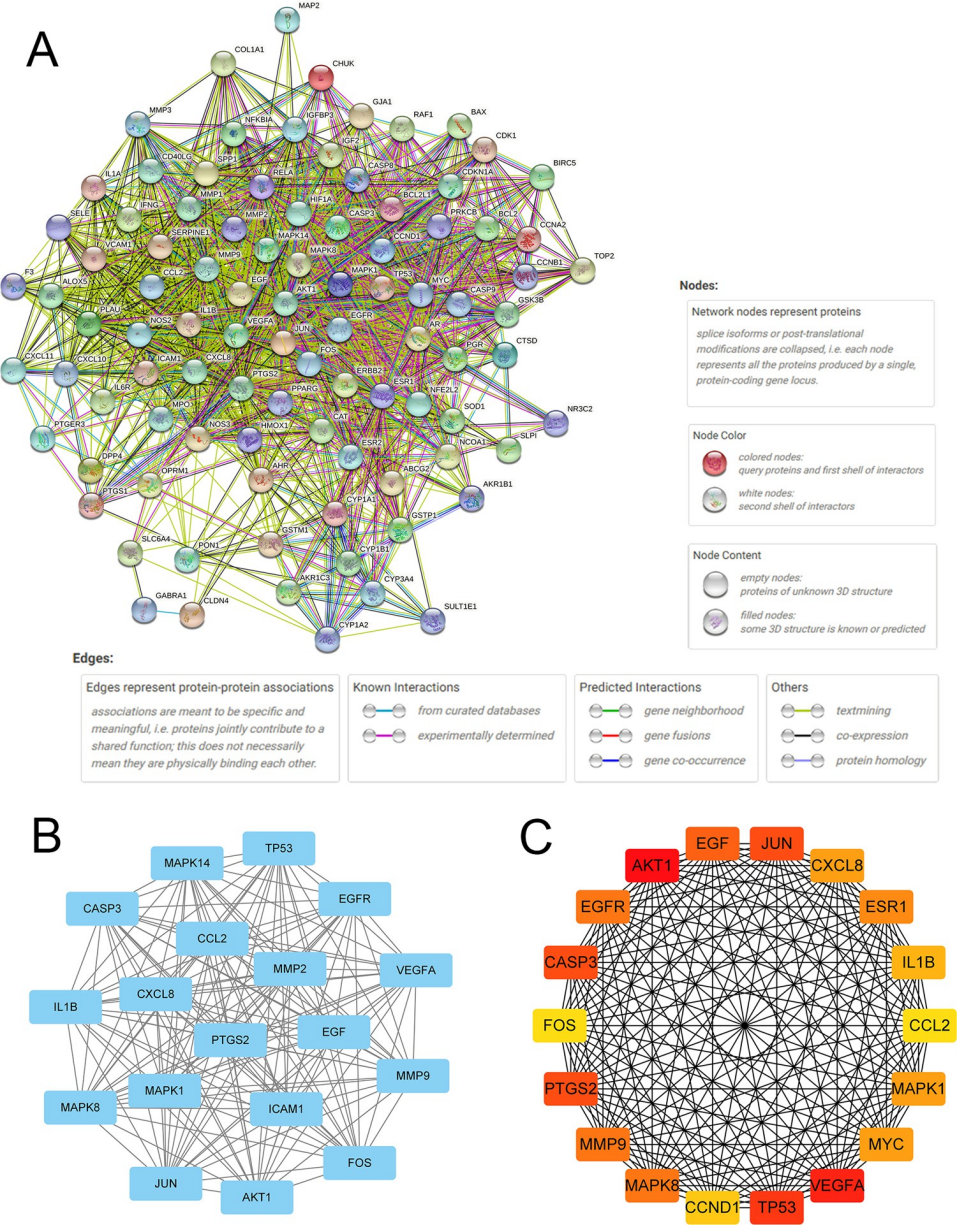
Protein–protein interaction (PPI) network and key subnetworks by Cytoscape. (A) PPI network exported from the STRING database and some annotations. (B) Key subnetwork screened after two filtrations using CytoNCA. (C) Key subnetwork of the top 18 nodes analyzed by CytoHubba.

The PPI network derived from the STRING database showed that the proteins encoded by these target genes had complex interactions (Fig 5A). We imported the PPI network into Cytoscape for further analysis. Finally, two key subnetworks were obtained by using CytoNCA and CytoHubba (Fig 5B and 5C).

### Results from MalaCards

We searched MalaCards using the key term of “endometriosis” and obtained 74 genes, 29 GO terms, and 3 pathways related to endometriosis. Among them, 15 genes were included in the abovementioned gene set, which was obtained by intersecting the QUF targets with the endometriosis-related gene set (Table 2). Moreover, 7 of the 24 biological processes in MalaCards were also found in the enrichment analysis results obtained above (Table 3). Among the 166 KEGG pathways mentioned above, “MicroRNAs in cancer” was also found in MalaCards and is thought to be closely related to endometriosis.

**Table 2:** Genes found in both the compound-target network and MalaCards.

Symbol	Description	Score
***PGR***	Progesterone Receptor	25.95
***ESR1***	Estrogen Receptor 1	24.48
***VEGFA***	Vascular Endothelial Growth Factor A	23.24
***ESR2***	Estrogen Receptor 2	23.09
***CXCL8***	C-X-C Motif Chemokine Ligand 8	21.17
***CCL2***	C-C Motif Chemokine Ligand 2	20.66
***IL1B***	Interleukin 1 Beta	20.03
***PTGS2***	Prostaglandin-Endoperoxide Synthase 2	19.73
***GSTM1***	Glutathione S-Transferase Mu 1	19.54
***MMP2***	Matrix Metallopeptidase 2	19.32
***CYP1B1***	Cytochrome P450 Family 1 Subfamily B Member 1	18.58
***CYP1A1***	Cytochrome P450 Family 1 Subfamily A Member 1	18.3
***MMP3***	Matrix Metallopeptidase 3	18.25
***MMP1***	Matrix Metallopeptidase 1	17.62
***PLAU***	Plasminogen Activator, Urokinase	16.34

**Table 3:** Biological processes identified in both the enrichment analysis results and MalaCards.

GO ID	Name	Score
**GO:0045766**	Positive regulation of angiogenesis	9.83
**GO:0030336**	Negative regulation of cell migration	9.81
**GO:0043536**	Positive regulation of blood vessel endothelial cell migration	9.72
**GO:1905563**	Negative regulation of vascular endothelial cell proliferation	9.63
**GO:0050728**	Negative regulation of inflammatory response	9.63
**GO:0006940**	Regulation of smooth muscle contraction	9.61
**GO:0016525**	Negative regulation of angiogenesis	9.43

### Component-target-pathway network construction

**Fig 6:**
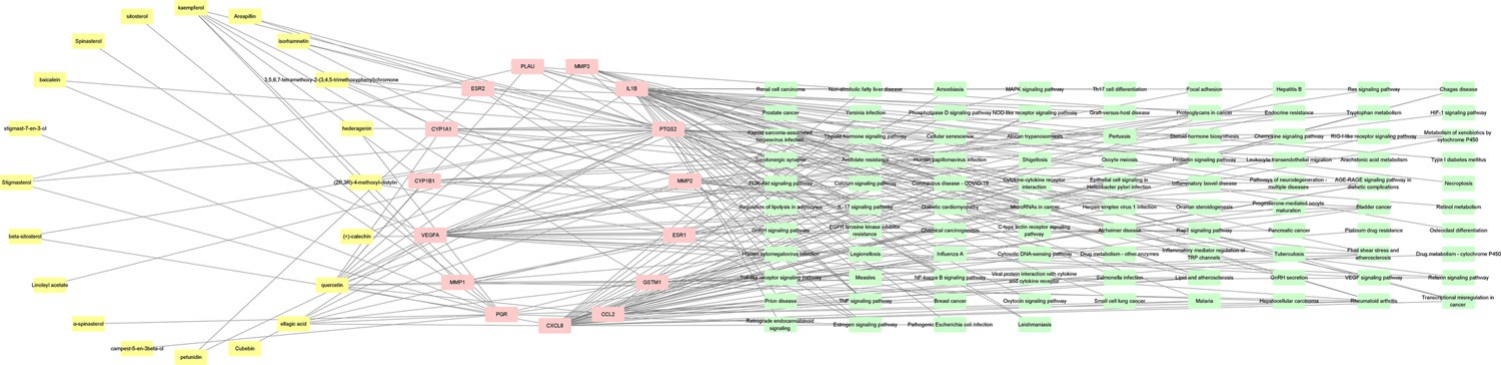
Component-target protein-pathway network. From left to right, the components of QUF, target proteins, and pathways are represented by the yellow, pink, and light green rounded rectangles, respectively. The edges show the connections between active compounds-protein and protein-pathway.

A component-target-pathway interaction network was established based on network pharmacology integration as shown in Fig 6. The network consisted of chemical components, protein targets, and pathways, and it includes 129 nodes and 248 edges. Fifteen target proteins with high scores in MalaCards interacted with 20 active components, and they were involved in 94 pathways. Among them, quercetin had 12 connections with genes highly related to endometriosis and seemed to be highly associated with endometriosis. The targets of quercetin were related to the inflammatory response, apoptosis and proliferation, and angiogenesis.

### Molecular docking

**Fig 7:**
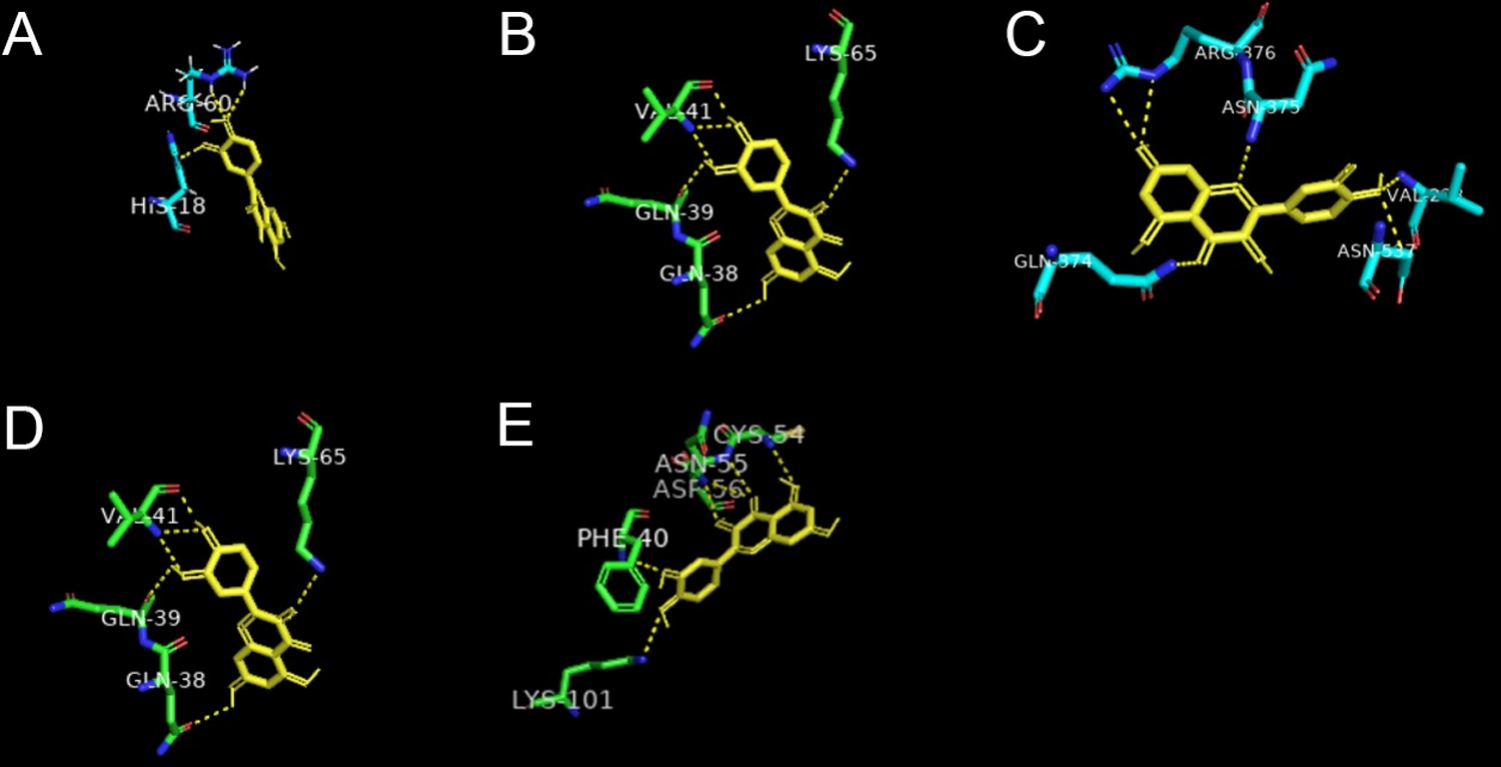
Molecular models of quercetin binding to its predicted protein targets. Proteins (A) *CCL2*-3IFD, (B) *CXCL8-*5WDZ, (C) *PTGS2*-5F1A, (D) *IL1B-*5I1B, and (E) *VEGFA*-3QTK interact with a quercetin molecule, and these interactions are represented by a yellow stick model. Lines represent residues in the binding sites. The yellow dashed lines represent hydrogen bonds. The abbreviation of amino acids and their positions on proteins are indicated next to the bonds.

Among the top genes in the two key subnetworks, *VEGFA*, *CXCL8*, *CCL2*, *IL1B* and *PTGS2* were also considered to be highly related to endometriosis in MalaCards. Therefore, protein 3QTK encoded by *VEGFA*, protein 5WDZ encoded by *CXCL8*, protein 3IFD encoded by *CCL2*, protein 5I1B encoded by *IL1B*, and protein 5F1A encoded by *PTGS2* were selected to conduct molecular docking. Three, two, one, one, and fifteen active compounds targeting *VEGFA*, *CXCL8*, *CCL2*, *IL1B*, and *PTGS2* proteins respectively were obtained from the compound-target interaction network. All these active compounds could easily enter and bind the active pocket of these five proteins. The docking scores were recorded in Table 4. Quercetin, the most important compound in QUF, was considered to have binding potential with all these key proteins. Hydrogen bonding was the main form of interaction between quercetin and the five target proteins (Fig 7).

**Table 4:** The docking information of five target proteins with the corresponding compounds.

	*VEGFA*-3QTK	*CXCL8-*5WDZ	*CCL2-*3IFD	*IL1B-*5I1B	*PTGS2*-5F1A
**quercetin**	-8.4	-6.4	-6.2	-7.5	-9.7
**baicalein**	-8.1	/	/	/	-9.5
**ellagic acid**	-8.8	-6.3	/	/	/
**(+)-Anomalin**	/	/	/	/	-8.8
**(+)-catechin**	/	/	/	/	-8.9
**(2R,3R)-4-methoxyl-distylin**	/	/	/	/	-8.6
**3,5,6,7-tetramethoxy-2-(3,4,5-trimethoxyphenyl) chromone**	/	/	/	/	-7.8
**Areapillin**	/	/	/	/	-8.6
**beta-sitosterol**	/	/	/	/	-9.1
**Cubebin**	/	/	/	/	-10.3
**hederagenin**	/	/	/	/	-8.9
**isorhamnetin**	/	/	/	/	-9.3
**kaempferol**	/	/	/	/	-9.4
**Linoleyl acetate**	/	/	/	/	-5.9
**petunidin**	/	/	/	/	-8.9
**Stigmasterol**	/	/	/	/	-9.6

The values in this table are docking scores between targets and compounds. The unit of the docking score is kcal/mol.

### Effects of quercetin on the expression levels of *VEGFA*, *CXCL8*, *CCL2*, *IL1B and PTGS2* in hEM15A cells

**Fig 8:**
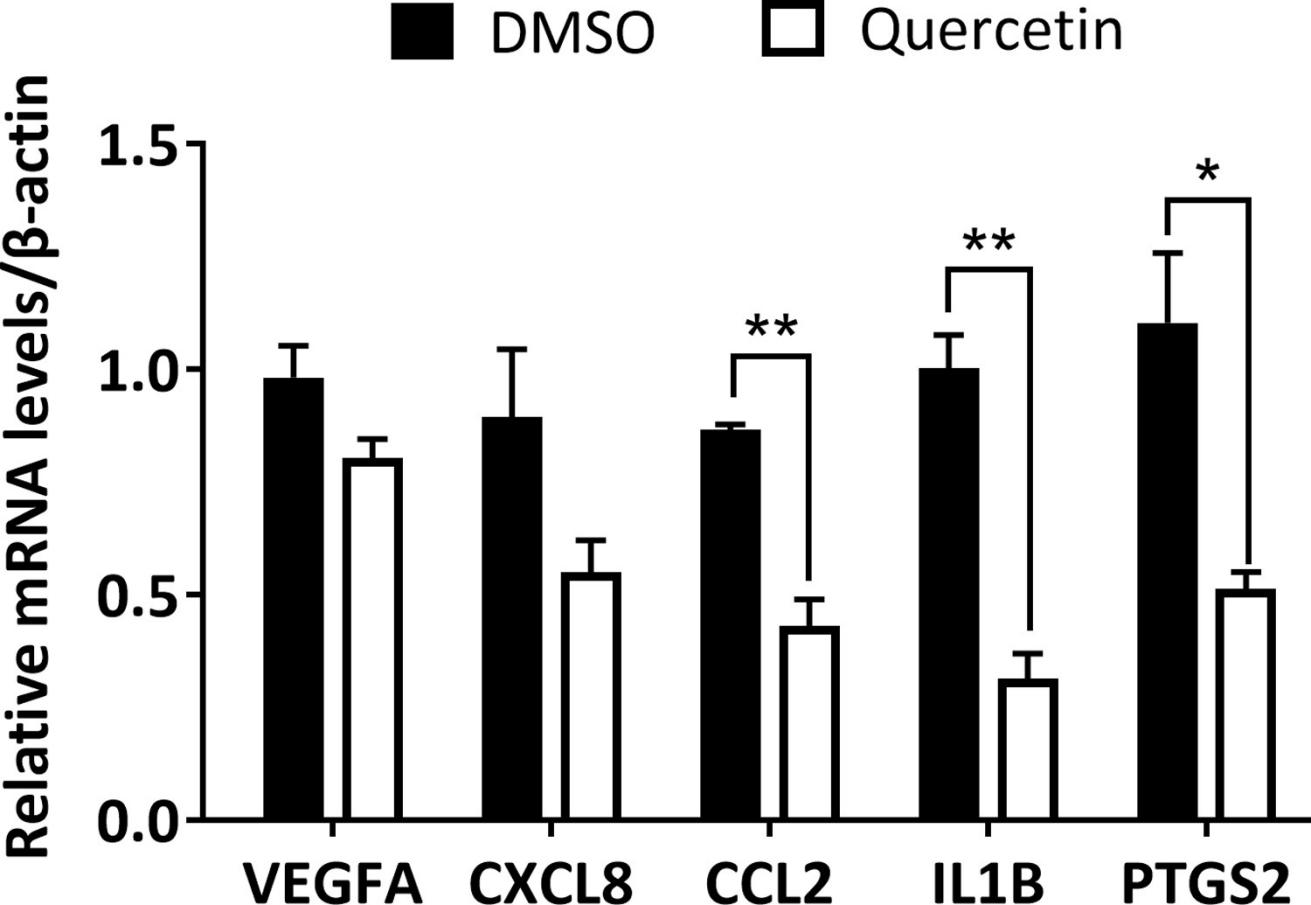
The effects of quercetin on the mRNA expression levels of *VEGFA*, *CXCL8*, *CCL2*, *IL1B and PTGS2* in hEM15A cells. **, P<0.01; *, P<0.05.

To further determine whether quercetin affects the expression of genes highly related to endometriosis, the mRNA expression levels of *VEGFA*, *CXCL8*, *CCL2*, *IL1B* and *PTGS2* in hEM15A cells were detected using RT–qPCR. The mRNA expression levels of *CCL2* (P < 0.01), *IL1B* (P < 0.01) and *PTGS2* (P < 0.05) in the quercetin group were significantly lower than those in the control group (Fig 8). However, no significant differences in the mRNA expression of *VEGFA* and *CXCL8* were detected between the quercetin group and the control group (P > 0.05). These results indicate that quercetin could significantly decrease the mRNA expression levels of *CCL2*, *IL1B* and *PTGS2* in hEM15A cells.

### Molecular dynamics simulation

**Fig 9:**
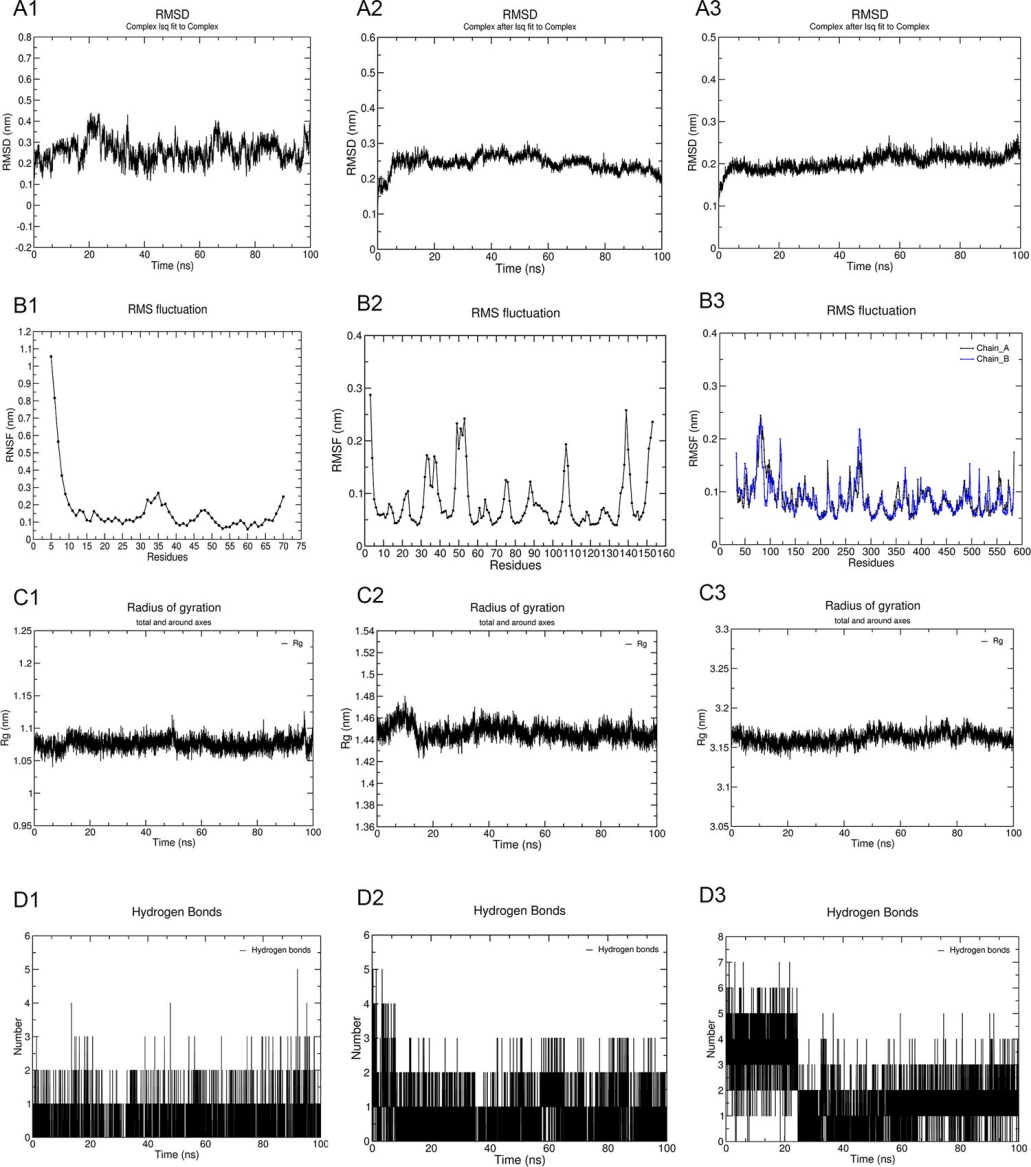
The results of molecular dynamics simulations. (A1-3) The RMSD of CCL2-quercetin, IL1B-quercetin and PTGS2-quercetin. (B1-3) The RMSF of CCL2-quercetin, IL1B-quercetin and PTGS2-quercetin. (C1-3) The Rg of CCL2-quercetin, IL1B-quercetin and PTGS2-quercetin. (D1-3) Number of hydrogen bond interactions of CCL2-quercetin, IL1B-quercetin and PTGS2-quercetin.

MD simulations of CCL2-quercetin, IL1B-quercetin and PTGS2-quercetin were performed for 100 ns each. The RMSD curve represents the stability of the protein conformation. The average RMSD was 0.25 nm, 0.24 nm and 0.20 nm, and the maximum RMSD was 0.44 nm, 0.31 nm and 0.27 nm for the CCL2-quercetin complex, IL1B-quercetin complex and PTGS2-quercetin complex, respectively. The PTGS2-quercetin complex showed a minimum deviation pattern, whereas the CCL2-quercetin complex showed the highest deviation pattern (Fig 9A1-9A3). RMSD presented certain fluctuations in the early stage, and the PTGS2-quercetin complex first attained stability after 5.2 ns (Fig 9A3). RMSF was calculated to interpret the fluctuations of the protein structures at the residue level. The residue fluctuations were all less than 0.3 nm in the three complexes (Fig 9B1-9B3), and the CCL2-quercetin complex had minimal fluctuations when compared with the other two complexes. The Rg curve represents the compactness of the overall structure of the protein. On average in Rg, the highest deviation pattern was observed in the PTGS2-quercetin complex, which may be induced by the dimeric structure and large molecular weight of PTGS2 (Fig 9C3). However, the other two complexes presented a lower deviation pattern (Fig 9C1-9C2). These findings suggest that the protein conformations were stable and that the binding of quercetin did not affect protein folding. The hydrogen bonds that formed show the strength of the binding between the ligand and protein. The PTGS2-quercetin complex had the largest number of hydrogen bonds, while the CCL2-quercetin complex had the fewest hydrogen bonds (Fig 9D1-9D3). The stability of the system is mainly accounted for on the basis of RMSD values and hydrogen bonding results. The results of the MD simulation indicated that the combinations of quercetin and the proteins were relatively stable.

## Discussion

Network pharmacology is a systematic research method that is suitable for the ‘‘multicomponent, multitarget, multipathway” synergistic characteristics of TCM. In this study, we explored the possible therapeutic mechanism of a patented Chinese herbal medicine for treating endometriosis based on network pharmacology, molecular docking, cell experiments, and molecular dynamics simulation methods. This study may provide directions for further experimental study on the molecular mechanisms of TCM in treating endometriosis.

GZFLW is a classical prescription for endometriosis in TCM and consists of Cinnamomi Ramulus (Guizhi), Poria Cocos (Fuling), Persicae Semen (Taoren), Radix Paeoniae Rubra (Chishao) and Cortex Moutan (Mudanpi). This prescription can be applied to treat endometriosis based on its anti-inflammatory, anticoagulant, analgesic, immune function-regulating, and blood circulation-promoting effects [7].

When compared with GZFLW [8], we found many similar active compounds in QUF and GZFLW. The main compounds in QUF, such as quercetin, kaempferol, baicalein, beta-sitosterol, stigmasterol, ellagic acid, hederagenin, and (+)-catechin, were also included in GZFLW. In addition, isorhamnetin from Radix Bupleuri was unique in QUF. It had 15 targets in the endometriosis-related gene set, and some of these targets, such as *ESR1*, *ESR2 and PTGS2*, had a high correlation score in MalaCards. However, some hub compounds, such as pachymic acid, taxifolin, and campesterol in GZFLW, could not be found in QUF. Most of the hub targets in QUF shown in Fig 5B could be found in GZFLW, except *CASP3*, *CXCL8*, *and MAPK14*. Proteins encoded by these genes play important roles in cell apoptosis, the inflammatory response, proliferation and many other cellular processes, and these processes have been considered as the key effects by which TCM contributes to treating endometriosis [17].

In the present study, KEGG pathway enrichment analysis indicated that the main enriched pathways were in the KEGG class of endocrine and metabolic diseases, signal transduction, drug resistance, immune system, cell growth and death, and cancers. The pathways of QUF in treating endometriosis might be associated with the immune response, apoptosis and proliferation, oxidative stress, and angiogenesis. The AGE−RAGE signaling pathway in diabetic complications and the HIF−1 signaling pathway were also discovered in GZFLW and thought to be important in oxidative stress and angiogenesis [8,18,19]. MicroRNAs in cancer, one of the three pathways closely related to endometriosis in MalaCards, were also discovered in our study. MicroRNAs are involved in many biological aspects shared by ovarian cancer and endometriosis, such as proliferation, inflammation, inhibition of apoptosis, deregulation of angiogenesis and invasion of surrounding tissue [20]. The results of the GO analysis indicated that “response to oxidative stress” was one of the most significant terms in the BP category, and “transcription factor binding” was one of the most significant terms in the MF category.

*VEGFA* is the most potent and specific angiogenic factor, and it can increase cell proliferation, cell migration, and vascular permeability in endometriosis [21,22]. Studies have shown that gonadotropin releasing hormone (GnRH) analogs could reduce the production of *VEGFA* in eutopic endometrial cell cultures [23]. In the present study, we found that quercetin, baicalein, and ellagic acid in QUF all had high docking scores with *VEGFA* in our docking results. These findings might suggest a mechanism by which QUF could inhibit the development of endometriosis.

Cyclooxygenase 2 (COX-2), encoded by *PTGS2*, plays an important role in the development of endometriosis [24]. COX-2 was overexpressed in the endometriotic tissues of patients with endometriosis and can cause high cell proliferation, low levels of apoptosis, high invasion, angiogenesis, endometriosis-related pain and infertility [25]. Nonsteroidal anti-inflammatory drugs (NSAIDs), type II gonadotropin releasing hormone (GnRH II), dienogest (DNG), glycyrrhizin, and puerarin can inhibit COX-2 expression in different ways and are used as anti-endometriosis drugs [24]. In this study, 15 active compounds in QUF were analyzed using molecular docking with *PTGS2*-5F1A, and all of them had a high docking score. This demonstrates that QUF is likely to affect the multiple biological functions of COX-2 through a multi-ingredient synergistic mechanism.

The proteins encoded by *CXCL8*, *CCL2*, and *IL1B* are important mediators of the inflammatory response. Interleukin-8 (IL-8) and monocyte chemotactic protein 1 (MCP-1), encoded by *CXCL8* and *CCL2* respectively, showed higher expression levels in the eutopic endometrium of women with endometriosis [26]. Higher expression of interleukin-1beta (IL1beta) was also found in the endometrium from endometriosis patients [27]. In this study, we found that quercetin in QUF had high docking scores with these target proteins.

After administration of quercetin, the mRNA expression of *CCL2*, *IL1B* and *PTGS2* decreased significantly in hEM15A cells. However, no significant differences existed in either *VEGFA* or *CXCL8* expression. MD simulation was carried out to evaluate the stability and interaction of quercetin and its target proteins. During the 100 ns MD simulation, there was no significant change in the complex conformation, indicating that the binding of quercetin remained relatively stable. These results partly validated the docking results of quercetin and its target proteins, indicating the therapeutic effects of QUF on endometriosis.

There are still some limitations in our study. First, only one active compound in QUF was validated. More biological experiments are still needed to prove the “multicomponent, multitarget, multipathway” characteristics of QUF in treating endometriosis. Second, because all the databases are based on the available research results, the discovery of new targets related to endometriosis may be limited, and any updates may not always be timely. Moreover, it seems that the traditional network pharmacology approach can only analyze protein-coding genes. Other categories of genes that may also have important roles in treating endometriosis were not included in the analysis.

In conclusion, this study presents a high-throughput and economic method of identifying drug targets and reveals that QUF may have a therapeutic effect on endometriosis through a multitarget mechanism. Our findings not only confirm the clinical effectiveness of QUF but also provide a foundation for further experimental study.

## Supporting information

S1 Table Information for 49 active ingredients. (DOCX)
