# Supplementary material for: A network pharmacology approach to explore active compounds and pharmacological mechanisms of a patented Chinese herbal medicine in the treatment of endometriosis
Source: PLoS One. 2022 Feb 7;17(2):e0263614. doi: 10.1371/journal.pone.0263614 (PMC8820622; doi:10.1371/journal.pone.0263614)
Supplement: S1 Table — (DOCX) [file pone.0263614.s001.docx]

**S1 Table. Information for 49 active ingredients.**

| Herb name | Mol ID | Compound | OB/% | DL | Endometriosis Targets |
| --- | --- | --- | --- | --- | --- |
| *Radix Paeoniae Rubra* (Chishao) | MOL001002 | ellagic acid | 43.06 | 0.43 | 14 |
|  | MOL001918 | paeoniflorgenone | 87.59 | 0.37 | 1 |
|  | MOL001921 | Lactiflorin | 49.12 | 0.8 | / |
|  | MOL001924 | paeoniflorin | 53.87 | 0.79 | 1 |
|  | MOL001925 | paeoniflorin_qt | 68.18 | 0.4 | / |
|  | MOL002714 | baicalein | 33.52 | 0.21 | 20 |
|  | MOL002776 | Baicalin | 40.12 | 0.75 | / |
|  | MOL000358 | beta-sitosterol | 36.91 | 0.75 | 14 |
|  | MOL000359 | sitosterol | 36.91 | 0.75 | 2 |
|  | MOL004355 | Spinasterol | 42.98 | 0.76 | 2 |
|  | MOL000449 | Stigmasterol | 43.83 | 0.76 | 8 |
|  | MOL000492 | (+)-catechin | 54.83 | 0.24 | 4 |
|  | MOL006990 | (1S,2S,4R)-trans-2-hydroxy-1,8-cineole-B-D-glucopyranoside | 30.25 | 0.27 | / |
|  | MOL006992 | (2R,3R)-4-methoxyl-distylin | 59.98 | 0.3 | 4 |
|  | MOL006994 | 1-o-beta-d-glucopyranosyl-8-o-benzoylpaeonisuffrone_qt | 36.01 | 0.3 | / |
|  | MOL006996 | 1-o-beta-d-glucopyranosylpaeonisuffrone_qt | 65.08 | 0.35 | / |
|  | MOL006999 | stigmast-7-en-3-ol | 37.42 | 0.75 | 1 |
|  | MOL007003 | benzoyl paeoniflorin | 31.14 | 0.54 | / |
|  | MOL007004 | Albiflorin | 30.25 | 0.77 | / |
|  | MOL007005 | Albiflorin_qt | 48.7 | 0.33 | / |
|  | MOL007008 | 4-ethyl-paeoniflorin_qt | 56.87 | 0.44 | / |
|  | MOL007012 | 4-o-methyl-paeoniflorin_qt | 56.7 | 0.43 | / |
|  | MOL007014 | 8-debenzoylpaeonidanin | 31.74 | 0.45 | / |
|  | MOL007016 | Paeoniflorigenone | 65.33 | 0.37 | / |
|  | MOL007018 | 9-ethyl-neo-paeoniaflorin A_qt | 64.42 | 0.3 | / |
|  | MOL007022 | evofolinB | 64.74 | 0.22 | / |
|  | MOL007025 | isobenzoylpaeoniflorin | 31.14 | 0.54 | / |
|  | MOL002883 | Ethyl oleate (NF) | 32.4 | 0.19 | / |
|  | MOL005043 | campest-5-en-3beta-ol | 37.58 | 0.71 | 1 |
| *Curcumae* *Rhizoma* (Ezhu) | MOL000296 | hederagenin | 36.91 | 0.75 | 4 |
|  | MOL000906 | wenjine | 47.93 | 0.27 | / |
|  | MOL000940 | bisdemethoxycurcumin | 77.38 | 0.26 | / |
| *Radix Bupleuri* (Chaihu) | MOL001645 | Linoleyl acetate | 42.1 | 0.2 | 2 |
|  | MOL002776 | Baicalin | 40.12 | 0.75 | / |
|  | MOL000449 | Stigmasterol | 43.83 | 0.76 | 8 |
|  | MOL000354 | isorhamnetin | 49.6 | 0.31 | 15 |
|  | MOL000422 | kaempferol | 41.88 | 0.24 | 31 |
|  | MOL004598 | 3,5,6,7-tetramethoxy-2-(3,4,5-trimethoxyphenyl) chromone | 31.97 | 0.59 | 4 |
|  | MOL004609 | Areapillin | 48.96 | 0.41 | 6 |
|  | MOL013187 | Cubebin | 57.13 | 0.64 | 2 |
|  | MOL004624 | Longikaurin A | 47.72 | 0.53 | / |
|  | MOL004628 | Octalupine | 47.82 | 0.28 | / |
|  | MOL004644 | Sainfuran | 79.91 | 0.23 | / |
|  | MOL004648 | Troxerutin | 31.6 | 0.28 | / |
|  | MOL004653 | (+)-Anomalin | 46.06 | 0.66 | 2 |
|  | MOL004702 | saikosaponin c_qt | 30.5 | 0.63 | / |
|  | MOL004718 | α-spinasterol | 42.98 | 0.76 | 2 |
|  | MOL000490 | petunidin | 30.05 | 0.31 | 6 |
|  | MOL000098 | quercetin | 46.43 | 0.28 | 78 |
